# Mcm10 coordinates the timely assembly and activation of the replication fork helicase

**DOI:** 10.1093/nar/gkv1260

**Published:** 2015-11-17

**Authors:** Patricia Perez-Arnaiz, Irina Bruck, Daniel L. Kaplan

**Affiliations:** Florida State University College of Medicine, Department of Biomedical Sciences, Tallahassee, FL 32306, USA

## Abstract

Mcm10 is an essential replication factor that is required for DNA replication in eukaryotes. Two key steps in the initiation of DNA replication are the assembly and activation of Cdc45–Mcm2–7-GINS (CMG) replicative helicase. However, it is not known what coordinates helicase assembly with helicase activation. We show in this manuscript, using purified proteins from budding yeast, that Mcm10 directly interacts with the Mcm2–7 complex and Cdc45. In fact, Mcm10 recruits Cdc45 to Mcm2–7 complex *in vitro*. To study the role of Mcm10 in more detail *in vivo* we used an auxin inducible degron in which Mcm10 is degraded upon addition of auxin. We show in this manuscript that Mcm10 is required for the timely recruitment of Cdc45 and GINS recruitment to the Mcm2–7 complex *in vivo* during early S phase. We also found that Mcm10 stimulates Mcm2 phosphorylation by DDK *in vivo* and *in vitro*. These findings indicate that Mcm10 plays a critical role in coupling replicative helicase assembly with helicase activation. Mcm10 is first involved in the recruitment of Cdc45 to the Mcm2–7 complex. After Cdc45–Mcm2–7 complex assembly, Mcm10 promotes origin melting by stimulating DDK phosphorylation of Mcm2, which thereby leads to GINS attachment to Mcm2–7.

## INTRODUCTION

The initiation of DNA replication is a highly regulated process in eukaryotes. Initiation of DNA replication begins with the recruitment of the replicative helicase Mcm2–7 to origins of replication. The Mcm2–7 complex is loaded as an inactive head-to-head double hexamer which encircles dsDNA during late M phase and early G_1_ (1–3). This first step is called licensing and in *Saccharomyces cerevisiae* it requires the action of ORC1–6, Cdc6 and Cdt1 in an ATP-dependent reaction (4,5). Upon entry into S phase, two kinases are crucial for the activation of the replicative helicase: Dbf4-dependent kinase Cdc7 (DDK) and S phase cyclin-dependent kinase (S-CDK). Both kinases promote the activation of the helicase by recruiting Cdc45 and GINS to form the active CMG (Cdc45–Mcm2–7-GINS) complex (6,7). In budding yeast, other initiation proteins are involved in this process, including Sld2, Sld3 and Dpb11, but these initiation proteins do not travel with the replication fork (8–11). Also, during S phase, the Mcm2–7 complex is remodeled from the dsDNA-bound double hexamer to the ssDNA-bound single hexamer (12,13). Mcm2–7 may open at the Mcm2-Mcm5 interface allowing the extrusion of the lagging strand. This event may be triggered by phosphorylation of Mcm2 by DDK (14). Finally, the active form of the replicative helicase is a single hexamer in complex with Cdc45 and GINS that encircles only the leading strand DNA (6,12,13,15,16).

Mcm10 is an evolutionary conserved protein required for DNA replication in eukaryotes (17,18), but its exact contribution still remains controversial (19). Mcm10 is not related in primary sequence to the Mcm2–7 proteins and does not have any enzymatic activity (20). Mcm10 shows ssDNA and dsDNA binding activities *in vitro* (21–26) and associates with chromatin at the G_1_/S phase transition (19,27,28). Studies in budding, fission yeast, *Xenopus, Drosophila* and humans showed that Mcm10 is able to interact with several proteins involved in DNA replication initiation including the Mcm2–7 complex (18,27,29–33) and Cdc45 (34–36). Work in multiple model organism from different laboratories suggested that Mcm10 could be involved in the recruitment of Cdc45 to the Mcm2–7 complex (28,36–38). However, recent studies in budding and fission yeast showed that a stable CMG complex forms in the absence of Mcm10 (39–42). We sought to resolve this conflict in this manuscript. Moreover, Mcm10 may be playing an active and essential role in promoting origin DNA melting and ssDNA extrusion from the Mcm2–7 complex central channel since CMG complex is inactive and is not able to unwind DNA in the absence of Mcm10 (28,40–42); however, the mechanism for Mcm10-stimulated helicase activation is not known (19). We also sought to determine the mechanism underlying Mcm10-stimulated origin melting in this manuscript.

The amount of Mcm2–7 double hexamer complexes loaded onto DNA to license replication during late M and G_1_ is significantly in excess over the number of Mcm2–7 complexes that are activated during S phase (43). However, initiation factor (Sld3, Sld2, Dpb11, Mcm10, and Sld7) binding to a given Mcm2–7 double hexamer is somehow coordinated with DDK phosphorylation of the same Mcm2–7 double hexamer, leading to the recruitment of Cdc45 and GINS to that particular Mcm2–7 double hexamer. Thus, assembly and activation of the replication fork helicase are coordinated in a manner that is not presently understood. In this manuscript, we sought to elucidate a mechanism that coordinates helicase assembly with helicase activation.

We show herein with purified proteins that Mcm10 binds directly to the Mcm2–7 complex and Cdc45. Furthermore, Mcm10 can recruit Cdc45 to Mcm2–7 in a DDK-independent manner *in vitro*, while Sld3 participates in a DDK-dependent mechanism for Cdc45 recruitment *in vitro*. Cells lacking Mcm10 show a growth defect as a result of defective DNA replication. In the absence of Mcm10, Cdc45 and GINS interaction with Mcm2–7 is delayed and RPA interaction with origin DNA is reduced during S phase *in vivo*. In addition, Mcm2 phosphorylation by DKK is reduced in the absence of Mcm10 *in vivo* and Mcm10 is able to stimulate DDK phosphorylation of Mcm2 *in vitro*, supporting the role for Mcm10 in stimulating Mcm2 phosphorylation by DDK during S phase. We conclude that Mcm10 coordinates the assembly of the helicase with phosphorylation of the helicase, a critical mechanism to ensure that particular Mcm2–7 double hexamer complexes are assembled and activated in a coordinated manner.

## MATERIALS AND METHODS

### Yeast strains and cell growth

All yeast strains used in this study are listed in Supplementary Table S1.

The *mcm10–1-*aid strains were obtained from National BioResource Project-Yeast (NBRP-Yeast) and *mcm2-*td degron strain was obtained from Karim Labib (44).

In all experiments, cells were grown at 25°C to slow down replication dynamics as described before (45). Medium used in this study is YP medium (1% yeast extract, 2% peptone) supplemented with 2% of glucose (YPD) or galactose (YPG). To induce degradation of aid tagged proteins in auxin inducible degron, exponentially growing cells in YPD were transferred to YPG to induce the expression of OsTIR1–9Myc for 45 min. A natural auxin, indole-3-acetic acid (IAA) (Sigma), was then added to medium at a final concentration of 500 μM. *mcm2-*td strains carrying plasmids for exogenous expression of *wild-type mcm2*, *mcm2–2A*and *mcm2–2D* were grown overnight in minimal medium with 2% raffinose at 25°C. *Wild-type* and mutant Mcm2 proteins were overexpressed in YPG from galactose-inducible promoter at 37°C to induce the degradation of endogenous *MCM2* gene.

### Plasmids

The following plasmids were used in this study: pIB302 (pRS415 CEN6/ARSH4 GALS::MCM2 LEU2), IB305 (pRS415 CEN6/ARSH4 GALS::mcm2S164A, S170A LEU2) and IB306 (pRS415 CEN6/ARSH4 GALS::mcm2S164D, S170D LEU2).

### Yeast serial dilution analysis

Serial dilution was performed as described (46). Yeast strains in overnight culture were transferred to YPG containing 500 μM auxin and incubated for 2 h at 25°C. The 10-fold dilution was performed and spotted onto YPG plates under either permissive (-auxin) or restrictive conditions (+auxin) and incubated at 25°C for 3 days.

### Co-immunoprecipitation

*mcm10–1-*aid cells initially cultured in YPD were arrested in G_1_ phase in the presence of α-factor (Zymo Research) for 3 h. They were transferred to YPG containing α-factor and subsequently incubated for 45 min. The culture was split into two to further incubate in the presence or absence of 500 μM IAA for 1 h before being released into YPG after extensive washes. Time course samples were taken at the indicated points. *mcm2-*td cells grown overnight in minimal medium containing raffinose at 25°C were treated with α-factor for 3 h at 37°C in YPG media. Following extensive washes, cells were further incubated at 37°C for the indicated times. Co-immunoprecipitation was performed as described (47). Cells were collected and lysed at 4°C with acid-washed glass beads (Sigma) in IP buffer [100 mM HEPES–KOH pH 7.9, 100 mM potassium acetate, 10 mM magnesium acetate, 2 mM sodium fluoride, 0.1 mM Na_3_VO_4_, 20 mM β-glycerophosphate, 1% Triton X-100, 0.7 μg/ml pepstatin, 0.1 μg/ml leupeptine, 0.1 mM PMSF, 1× complete protease inhibitor cocktail without EDTA (Roche)] using a BeadBeater. Lysed material was treated with 200 U of Benzonase (Novagen) at 4°C for 30 min. Clarified extracts were then mixed with 2 μl of specific antibody and rotated for 2 h at 4°C. Following this, 50 μl of Protein G Sepharose beads (GE Healthcare) equilibrated in IP buffer were added to the extracts and further rotated for 1 h at 4°C. Beads were washed twice with IP buffer and finally resuspended in SDS-sample buffer. Western analysis was performed and blots were scanned using the LI-COR Odyssey Infrared Imager and analyzed and quantified in the Image Studio 4.0 Software.

### Chromatin immunoprecipitation

Cells initially cultured in YPD were arrested in G_1_ phase in the presence of α-factor (Zymo Research) for 3 h. They were transferred to YPG containing α-factor and subsequently incubated for 45 min. The culture was split into two to further incubate in the presence or absence of 500 μM IAA for 1 h before being released into YPG after extensive washes. Time course samples were taken at the indicated points. Chromatin immunoprecipitation was performed as described (48). Formaldehyde cross-linked cells were lysed with acid-washed glass beads in a BeadBeater. DNA was fragmented by sonication (Branson 450, 5 cycles of 15 s each). RPA (2 μl) antibody and magnetic protein A beads (Invitrogen) were added to the cleared lysate to immunoprecipitate DNA. Immunoprecipitates were then washed extensively to remove nonspecific DNA. Eluted DNA was subjected to PCR analysis using primers directed against *ARS306* or a midway between *ARS305* and *ARS306* as described (42). We performed PCR with [^32^P-α]-dCTP as a component of the PCR reaction to quantify the amplified product. The radioactive band in the native gel, representing specific PCR amplified DNA product, was quantified by phosphorimaging and normalized by a reference standard (a PCR reaction with a known quantity of template DNA) run in the same gel.

### Fluorescence activated cell sorting

Samples were fixed with 70% ethanol and FACS was performed as described previously (46). Cells were grown overnight in YPD at 25°C. For G_1_ arrest cells were treated with α-factor (Zymo research) for 3 h. They were transferred to YPG containing α-factor and subsequently incubated for 45 min. The culture was split into two to further incubate in the presence or absence of IAA before being released into YPG. Cells were collected at the indicated time intervals and stained with propidium iodide. Cell cycle progression data were obtained using BD FACS Canto Ruo Special Order System and analyzed using FACS Diva software.

### Cloning and purification of proteins

Full length Mcm10 PCR product was cloned into SpeI/XhoI sites of pET-41 vector and NdeI/XhoI sites of pET-33 vector to contain an N-terminal GST tag or a PKA tag respectively as described (49). The cloning of Mcm10 into pET-41 generates two-tagged protein (a GST tag at the N-terminus and a His tag at the C-terminus). Purification of GST-Mcm10 uses sequential nickel and glutathione resins. The details of GST-Mcm10 and PKA-Mcm10 are described (49). GST-Cdc45 was purified as described (48). Mcm2–7 proteins were purified and Mcm2–7 complex was assembled from recombinant subunits as described (50,51). Native GINS was purified as described (52). Sld3-PKA was purified as described (52). GST-Dbf4 and GST-Cdc7 were purified a described (50). Protein kinase A was a generous gift from Susan Taylor.

### DDK kinase assay

Kinase reactions were performed in a volume of 25 μl and contained 25 mM HEPES–NaOH pH 7.5, 1 mM DTT, 10 mM magnesium acetate, 75 mM sodium acetate, 5 mM ATP, 0.1 mg/ml BSA, 1 mM sodium fluoride, 0.1 mM Na_3_VO_4_, 5 μg Mcm2 and 50 ng DDK. The amount of Mcm10 added in each reaction is described in the figure. Reactions were incubated at 30°C for 1 h. Reactions were stopped by addition of SDS-sample buffer and analyzed by western blot using an antibody against Mcm2–164–phosphoserine-170-phosphoserine.

### Kinase labeling of proteins

Kinase labeling was performed as described (49). Proteins with a PKA tag at the N-terminus (Mcm10, Mcm2, Mcm3, Mcm4, Mcm5, Mcm6, Mcm7, Mcm2–7, Mcm2–7 2D and Sld3) were labeled in a reaction volume of 100 ul that contain 20 μM of PKA-tagged protein with 5 μg PKA in kinase reaction buffer (25 mM Tris–HCl pH 7.5, 5 mM DTT, 50 mM MgCl_2_ and 500 μCi [γ-^32^P]-ATP). The reaction was incubated at 30°C for 1 h.

### GST pulldown assay

The GST pulldown assays were performed as described (49). GST-tagged protein attached to Glutathione Sepharose (GE Healthcare) previously equilibrated with GST binding buffer (40 mM Tris–HCl pH 7.5, 100 mM NaCl, 0.1 mM EDTA, 10% glycerol, 0.1% Triton X-100, 1 mM DTT, 0.7 μg/ml pepstatin, 0.1 μg/ml leupeptine, 0.1 mM PMSF and 0.1 mg/ml BSA) was incubated with varying concentrations of radiolabeled protein in GST binding buffer in a final reaction volume of 100 μl. The reactions were incubated at 30°C for 10 min with gentle mix every few minutes. When the binding was complete, the reactions were shifted to room temperature and glutathione beads were allowed to settle. The supernatant was removed and the beads were washed two times with 500 μl GST binding buffer. After the last wash, beads were heated at 90ºC for 10 min in SDS-sample buffer (2% SDS, 4% glycerol, 4 mM Tris–HCl, 2 mM DTT and 0.01% bromophenol blue). The reactions were then analyzed by SDS-PAGE followed by phosphorimaging and quantification.

### Glycerol gradient ultracentrifugation

We performed the interaction of 60 pmol of Mcm10 with 30 pmol of Mcm2–7 complex in a buffer containing 30 mM Tris–HCl pH 7.5, 100 mM NaCl, 10% glycerol, 0.1% Triton, 0.1 mM DTT, 0.7 μg/ml pepstatin, 0.1 μg/ml leupeptine and 0.1 mM PMSF. This complex was then sedimented through a 4-ml linear 10–40% (v/v) glycerol gradient in a Beckman Coulter Optima MAX-XP Ultracentrifuge at 45 000 rpm (MLS-50 rotor, Beckman) for 18 h at 4°C. After centrifugation, the gradient was fractioned into 0.2-ml fractions from the bottom of the tube. These fractions were analyzed by SDS-PAGE. Western analysis was then performed and blots were scanned using the LI-COR Odyssey Infrared Imager and analyzed and quantified in the Image Studio 4.0 Software.

### Antibodies

Antibodies against Mcm10, Mcm2 and Mcm2-phosphoserine-164-phosphoserine-170-phosphoserine, Cdc45, GINS and Sld3 were supplied by Open Biosystems (we supplied the antigens). Crude serum was purified against immobilized antigen to remove nonspecific antibodies. The specificity of each antibody was analyzed by western blot of purified proteins and yeast extract. Antibodies directed against RPA (Thermo Scientific), FLAG epitope (Sigma), Arp3 (Santa Cruz Biotechnology) and His Tag (Thermo Scientific) were commercially purchased.

## RESULTS

### *mcm10-1-*aid cells exhibit cell growth and DNA replication defects

**Figure 1.**
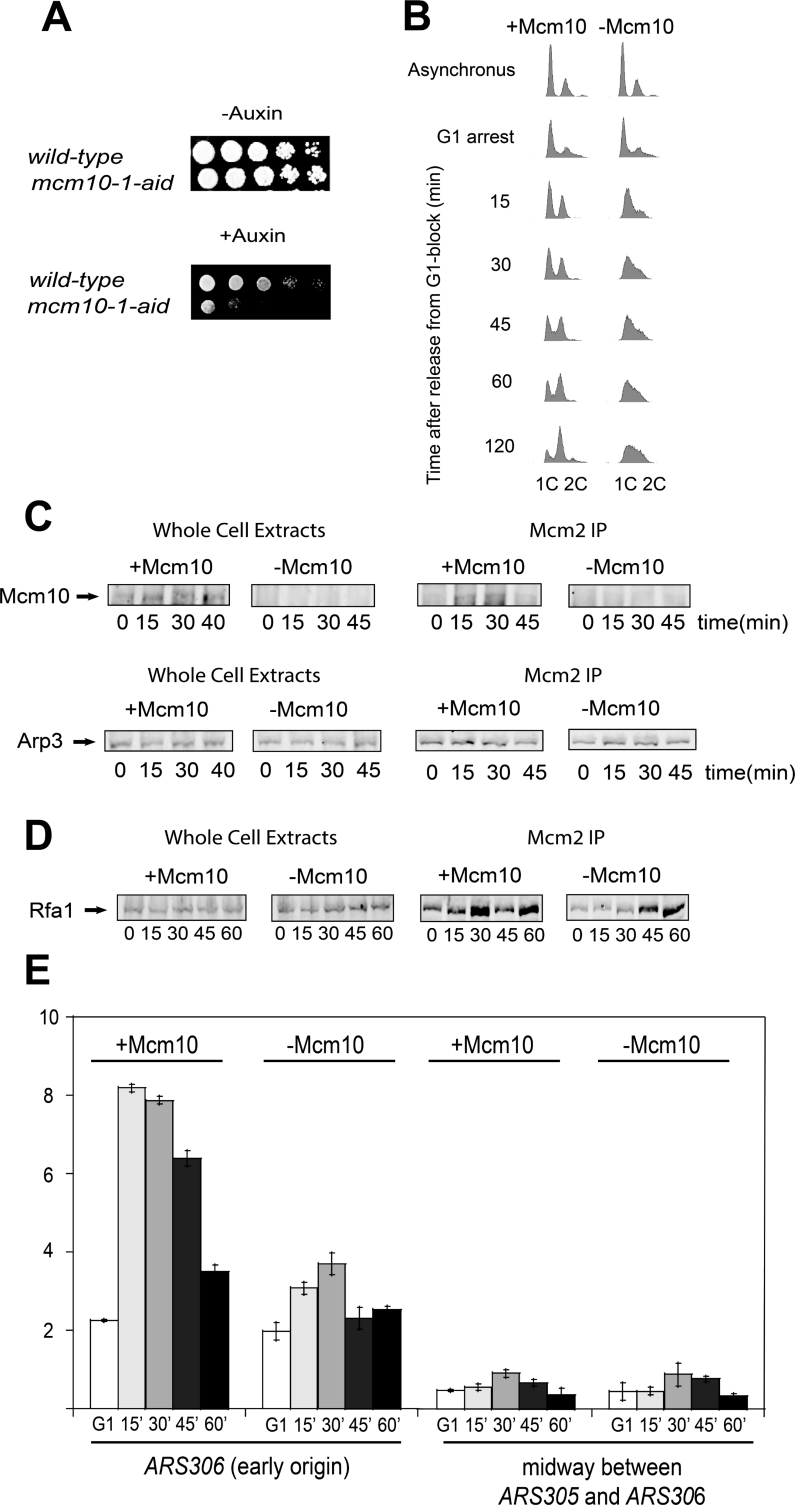
*mcm10-1-*aid cells exhibit a defect in DNA replication. Origin melting is defective in the absence of Mcm10. (**A**) Serially diluted *mcm10–1-*aid or control cells were spotted and grown on YPG plate under either permissive or restrictive conditions. Plates were incubated for 3 days at 25°C. (**B**) *mcm10-1-*aid cells were grown as described in Materials and Methods. Cells were analyzed by FACS with propidium iodide staining for DNA content. (**C**) *mcm10-1-*aid cells were grown as described in Materials and Methods. (**C**, **left panel**) Whole cell extracts were analyzed by western blot for expression of Mcm10. (**C**, **right panel**) Cells extracts were immunoprecipitated with antibodies directed against Mcm2 followed by western analysis with Mcm10 antibody. Whole cell extracts and immunoprecipitated samples were analyzed by western blot for Arp3 expression as a loading control. **D**) *mcm10-1-*aid cells were grown as described in Materials and Methods. (**D**, **left panel)** Whole cell extracts were analyzed by western blot for expression of Rfa1. (**D**, **right panel**) Antibodies against Mcm2 were used for immunoprecipitation followed by western analysis of Rfa1. (**E**) *mcm10-1-*aid cells were grown and chromatin immunoprecipitation was performed as described in Materials and Methods. Radioactive PCR bands were quantified, averaged and plotted. Graph represents mean values from three independent experiments and error bars indicate the standard deviation of the mean.

To investigate the role of Mcm10 *in vivo* we used an auxin-inducible degron (AID) system obtained from a published source (40,53), in which Mcm10 degradation is induced by the addition of auxin to the culture media before releasing cells from G_1_. We performed a 10-fold serial dilution and spotted control and *mcm10-1-*aid cells onto YPG plates in the absence (permissive conditions) and in the presence (restrictive conditions) of auxin. At the permissive conditions, control and *mcm10-1-*aid cells grow to similar levels. However, at the restrictive conditions, *mcm10-1-*aid cells show a substantial growth defect compared to control cells (Figure 1A) as described previously (40).

We further studied cell cycle progression into S phase using FACS analysis to determine whether the growth defect in *mcm10-1-*aid cells was the result of a DNA replication defect. Cells expressing Mcm10 exhibited normal progression through S phase with an accumulation of 2C DNA at 60 min after α-factor release. However, cells in which Mcm10 was depleted exhibited a substantial defect in progression into S phase (Figure 1B) as described previously (40). These data suggest that cells show a defect in DNA replication in the absence of Mcm10.

We next wanted to study the interaction between the Mcm2–7 complex and Mcm10 *in vivo*. We arrested cells in G_1_ and then cells were transferred to YPG with α-factor for 45 min and incubated them under permissive and restrictive conditions for 1 h before releasing them into S phase for the indicated times. No cross-linking agent and no hydroxyurea were used in these experiments. We made whole cell extracts and precipitated them with antibodies against Mcm2 to isolate Mcm2–7 complexes loaded onto origins of replication. We next probed these immunoprecipitates with Mcm10 antibody, so Mcm10-Mcm2 co-IP signal reflects the interaction between Mcm10 and loaded Mcm2–7. In cell extracts treated under restrictive conditions no Mcm10 was detected (Figure 1C), as it was expected and published by a different group (40). When immunoprecipitated cells were probed with Mcm10 antibody, we were able to detect Mcm10 bound to the Mcm2–7 complex at 15 and 30 min (Figure 1C, right panel), indicating that Mcm10 associates with Mcm2–7 during S phase. There is a decrease in Mcm2–Mcm10 interaction at 45 min, suggesting that the interaction between Mcm2–7 and Mcm10 is restricted to S phase.

### Origin melting is delayed in the absence of Mcm10

We next determined whether origin melting/unwinding is defective in the absence of Mcm10. For this purpose, we analyzed the association of Rfa1, the largest subunit of the yeast single-stranded DNA binding protein Replication Protein A (RPA), to origins of replication. Cells expressing Rfa1–5FLAG in the *mcm10–1-*aid background were grown as in Figure 1C. We made whole cell extracts and then immunoprecipitated cells with an antibody directed against Mcm2 and probed them with Rfa1 antibody. In the presence of Mcm10 we were able to detect Rfa1 mainly at 15 and 30 minutes, indicating that origin melting occurs at this time during S phase (Figure 1D, right panel). However, Rfa1 association was substantially decreased at 15 and 30 minutes in the absence of Mcm10 (Figure 1D, right panel), suggesting that origin melting is delayed in the absence of Mcm10.

To confirm this result, we performed chromatin immunoprecipitation (ChIP) using antibodies against RPA. We grew *mcm10-1-*aid cells as in Figure 1C and release them into medium lacking α-factor for the indicated times. We then performed PCR using the immunoprecipitate as DNA template and oligonucleotides against the early origin *ARS306* or a non-origin region located midway between *ARS305* and *ARS306*. In the presence of Mcm10, Rfa1 association was detected at 15 min during S phase. In contrast, cells in which Mcm10 was depleted showed a substantially reduced Rfa1 association at this time point (Figure 1E). No increase in signal was observed for the non-origin sequence (Figure 1E). The decrease in RPA-ChIP signal at origin sequence supports the result shown in Figure 1D and described previously (40–42) and the importance of Mcm10 for origin melting/unwinding during the initiation of DNA replication.

### CMG complex formation is delayed in the absence of Mcm10

**Figure 2.**
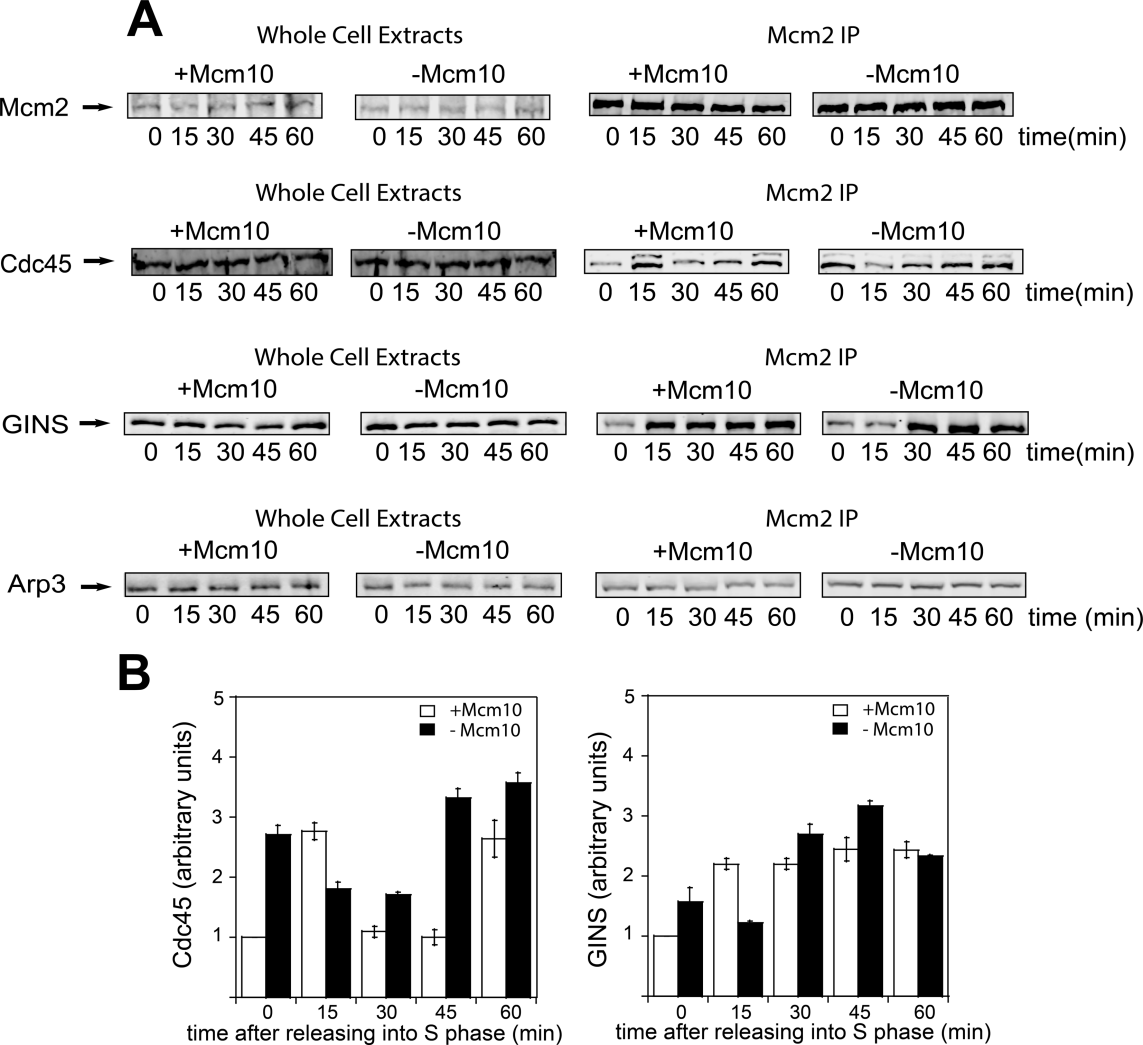
CMG complex formation is delayed in the absence of Mcm10. (**A)***mcm10-1-*aid cells were grown as described in Materials and Methods. (**A**, **left panel**) Whole cell extracts were analyzed by western blot for the expression of the indicated proteins. (**A**, **right panel**) Cells were immunoprecipitated with antibodies directed against Mcm2, followed by western analysis with antibodies directed against Mcm2, Cdc45 and GINS. Whole cell extracts and immunoprecipitated samples were analyzed by western blot for Arp3 expression as a loading control. (**B**) Results from experiments similar to those shown in (A) were quantified, averaged and plotted. Graphs represent mean values from three independent experiments and error bars indicate the standard deviation of the mean.

We wanted to study if Mcm10 plays a role during CMG complex assembly. For that purpose, we grew *mcm10-1-*aid cells as in Figure 1C and released them into medium lacking α-factor for the indicated times during S phase. No cross-linking agent and no hydroxyurea were used in these experiments to assess whether CMG complex is formed under physiological cellular conditions. Whole cell extracts were made and these cells were then precipitated using an antibody directed against Mcm2. Whole cell extracts exhibited similar levels of Mcm2, Cdc45 and GINS, suggesting that the absence of Mcm10 does not affect protein levels *in vivo* (Figure 2A, left panel). Levels of Mcm2 were equivalent for cells under permissive and restrictive conditions, indicating that equivalent levels of Mcm2 were immunoprecipitated in these experiments (Figure 2A, right panel). In cells under permissive conditions, Cdc45 and GINS associate at 15 min after entry into S phase, this result is consistent with their recruitment to replication origins during S phase, but both Cdc45 and GINS levels were significantly and substantially reduced in cells lacking Mcm10 at this same time point (Figure 2). However, at later time points we observe a similar or stronger level of Cdc45–GINS–Mcm2–7 interaction in cells lacking Mcm10. There is also an increase in Cdc45–GINS–Mcm2–7 interaction at time zero for cells lacking Mcm10 (Figure 2), suggesting that Mcm10 is also important to prevent the premature assembly of CMG in G_1_, for unknown reasons.

We also interrogated CMG assembly *in vivo* with a different approach, wherein we performed immunoprecipitation with antibodies against Cdc45 and GINS and probed them with Mcm2 antibody. At 15 min after releasing cells into S phase, the level of Mcm2 in both Cdc45 and GINS immunoprecipitates was reduced in cells lacking Mcm10 (Supplementary Figure S1). Again, at later time points we observe a similar or stronger amount of Cdc45 and GINS bound to Mcm2–7 in the absence of Mcm10. These results suggest that Mcm10 is required for Cdc45–GINS-Mcm2–7 recruitment upon entry into S phase and that the assembly of CMG complex is delayed in the absence of Mcm10. There is a discrepancy in our data for the 60-minute time points for the Mcm2–GINS interaction between our two experiments. We do not know the reason for this discrepancy at the present time.

### Mcm10 interacts with DDK and stimulates DDK phosphorylation of Mcm2

**Figure 3.**
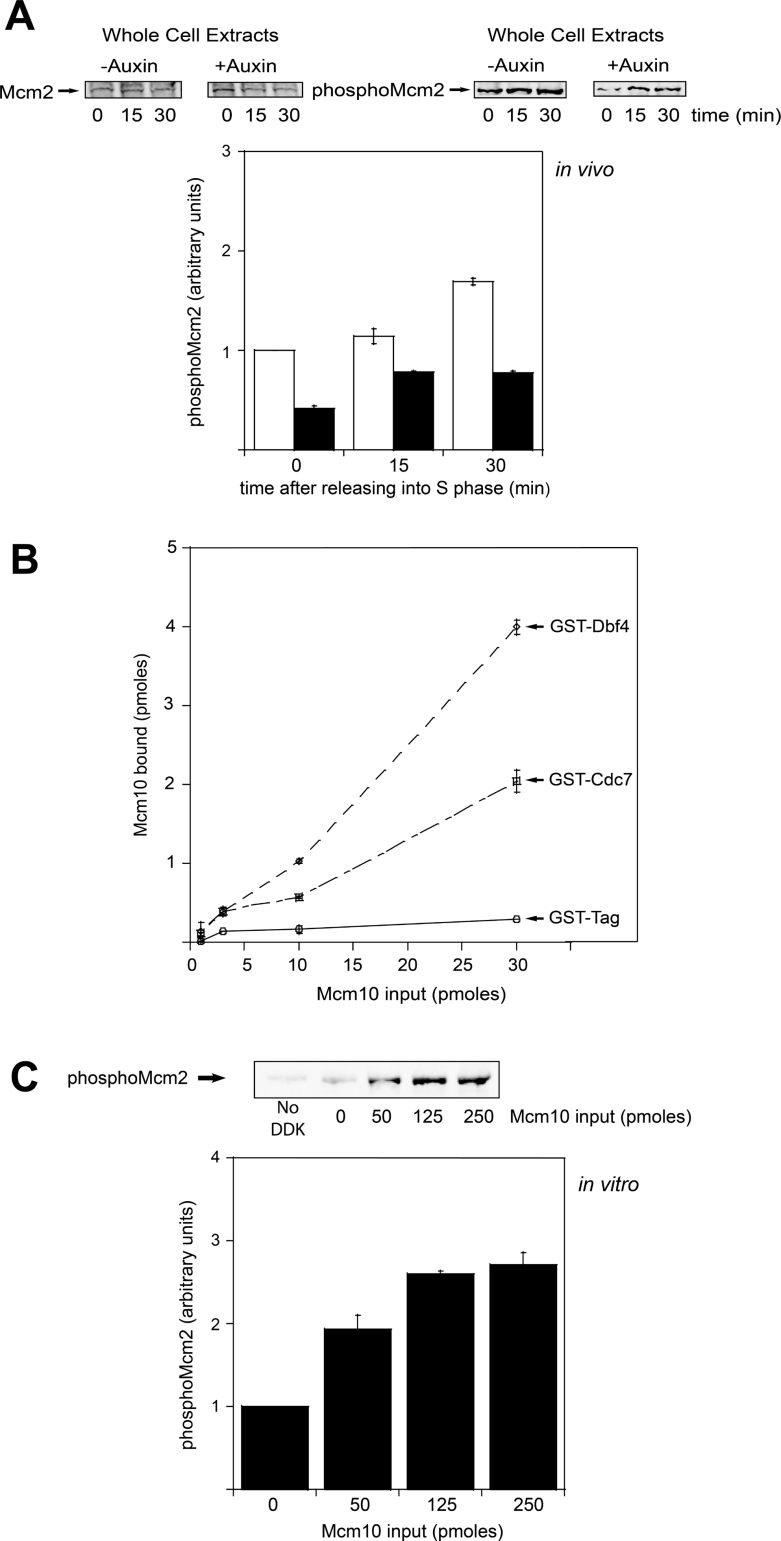
Mcm10 interacts with DDK and stimulates Mcm2 phosphorylation by DDK. (**A**) *mcm10-1-*aid cells were grown as described in Materials and Methods. (**A**, **left panel**) Whole cell extracts were analyzed by western blot for expression of Mcm2. (**A**, **right panel**) Whole cell extracts were analyzed by western blot for expression of phospho-Mcm2. Results from similar experiments were quantified, averaged and plotted. (**B**) 30 pmol of GST-Dbf4, GST-Cdc7 or GST tag was incubated with increasing concentrations of radiolabeled PKA-Mcm10 at 30°C for 10 min in a GST pulldown assay. Results from similar experiments were quantified, averaged and plotted. (**C**) 5 μg of Mcm2 was incubated with 50 ng of DDK and varying amounts of Mcm10 in a volume of 25 μl at 30°C for 1 h. The reactions were then analyzed by western blot for expression of phospho-Mcm2. Results from similar experiments were quantified, averaged and plotted. Graphs from (A), (B) and (C) represent mean values from two independent experiments and error bars indicate the standard deviation of the mean.

It is known that DDK phosphorylates Mcm2 during S phase (54) and that this phosphorylation may be responsible of opening the Mcm2–Mcm5 gate for the extrusion of ssDNA from the central channel of the Mcm2–7 ring (14). We used a validated phosphospecific antibody against a peptide of Mcm2 that was phosphorylated at Ser-164 and Ser-170 (Mcm2 phospho-antibody) (14). We made whole cell extracts from cells grown under permissive and restrictive conditions and then used Mcm2 phospho-antibody to determine whether Mcm10 has any effect on Mcm2 phosphorylation by DDK *in vivo*. In cells expressing Mcm10, we detected an increase in DDK phosphorylation of Mcm2 as cells entered and progressed through S phase (Figure 3A). In cells lacking Mcm10, we observed an increase in Mcm2 phosphorylation during S phase as well, but the amount of phosphorylated Mcm2 was reduced to nearly half compared to normal cells (Figure 3A), indicating that DDK phosphorylation of Mcm2 is modestly decreased *in vivo* in the absence of Mcm10.

We next investigated the interaction between Mcm10 and DDK *in vitro*. For that purpose, we purified Mcm10, Dbf4 and Cdc7. Increasing amounts of radiolabeled Mcm10 were incubated with GST tag alone, GST-Dbf4 or GST-Cdc7 in a GST pulldown assay. We found that GST-Dbf4 binds tightly to Mcm10. In contrast, GST-Cdc7 binds weakly to Mcm10 (Figure 3B).

Then, we wanted to examine Mcm10 for its ability to affect DDK activity *in vitro*. For that purpose, we performed a kinase assay in which Mcm2 was incubated with DDK in the presence and in the absence of varying amounts of Mcm10. We detected a substantial increase in Mcm2 phosphorylation by DDK as increasing concentration of Mcm10 were added to the reaction (Figure 3C). These data suggest that Mcm10 interacts with DDK *in vitro* and stimulates the phosphorylation of Mcm2 by DDK *in vivo* and *in vitro* and this activity might have a critical role *in vivo*.

### Mcm10 binds to the Mcm2–7 complex

**Figure 4.**
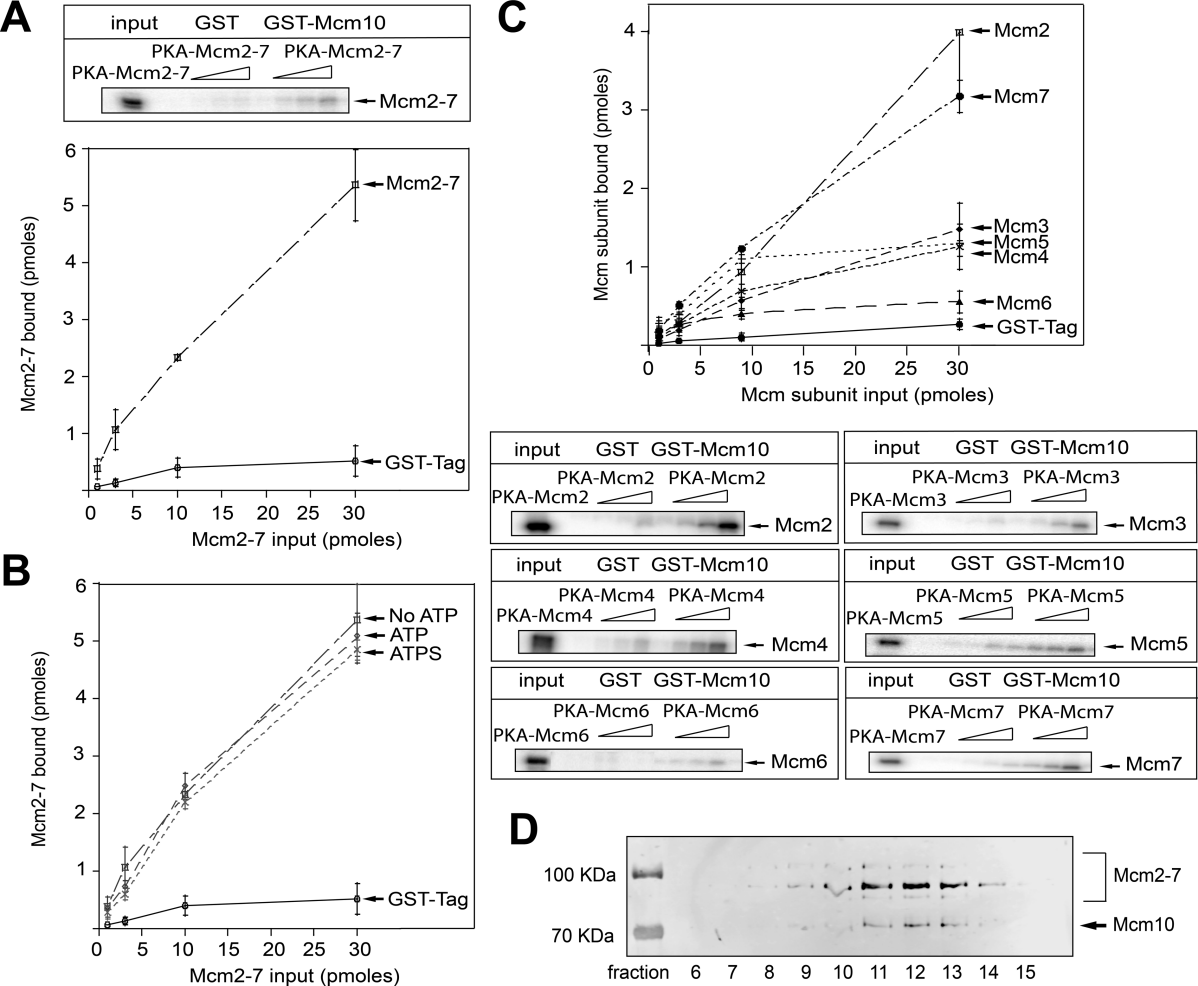
Mcm10 binds Mcm2–7 complex *in vitro*. (**A**) 30 pmol of GST-Mcm10 or GST tag was incubated with increasing concentrations of radiolabeled PKA-Mcm2–7 at 30°C for 10 min in a GST pulldown assay. Results from similar experiments were quantified, averaged and plotted. (**B**) 30 pmol of GST-Mcm10 was incubated with increasing concentrations of radiolabeled PKA-Mcm2–7 in a GST pulldown assay. These experiments were performed with 5 mM ATP, 5 mM ATPγS or no nucleotide. Results from similar experiments were quantified, averaged and plotted. (**C**) 30 pmol of GST-Mcm10 was incubated with increasing concentrations of radiolabeled PKA-Mcm2, PKA-Mcm3, PKA-Mcm4, PKA-Mcm5, PKA-Mcm6 or PKA-Mcm7 at 30ºC for 10 min in a GST-pulldown assay. Results from similar experiments were quantified, averaged and plotted. (**D**) Glycerol gradient ultracentrifugation analysis of the Mcm10–Mcm2–7 complex. The fractions were analyzed and quantified by western blot using anti-His Tag antibody. Graphs from (A), (B) and (C) represent mean values from two independent experiments and error bars indicate the standard deviation of the mean.

Activation of the eukaryotic helicase is a critical step in the initiation of DNA replication and Mcm10 has been shown to be indispensable for this process (19). We know that Mcm10 is able to interact with the Mcm2–7 complex *in vivo* (Figure 1C). Then, we purified Mcm10 and Mcm2–7 to investigate the interaction between these proteins *in vitro*. Varying concentrations of the radiolabeled Mcm2–7 complex were incubated with GST tag alone or GST-Mcm10 in a GST pulldown assay. We found that GST-Mcm10 is able to pull down radiolabeled Mcm2–7, more than 10 times the signal for GST alone (Figure 4A). These data suggest that Mcm10 binds directly to the Mcm2–7 complex. We also performed the experiment in the presence of 5 mM ATP, 5mM non-hydrolyzable ATPγS or no nucleotide to determine the influence of nucleotides on the interaction between Mcm10 and Mcm2–7. We could not detect any difference, suggesting that nucleotide binding does not affect the interaction between Mcm10 and Mcm2–7 (Figure 4B). We next investigated whether Mcm10 binds directly to every Mcm2–7 subunit individually. We incubated GST-Mcm10 with each purified and radiolabeled Mcm2–7 subunit. GST-Mcm10 is able to pull down Mcm2, Mcm3, Mcm4, Mcm5 and Mcm7 subunits with highest affinity for Mcm2 followed by Mcm7 (Figure 4C). Binding of Mcm6 was equivalent to GST background (Figure 4C). This result is not consistent with previous studies in which Mcm10 was found to interact with Mcm6 (18,42) and the discrepancy may be explained by our different experimental approach.

We also wanted to determine the stoichiometry of Mcm10 binding to the Mcm2–7 complex. For that purpose, we performed a glycerol gradient ultracentrifugation analysis. Most of the Mcm10–Mcm2–7 complex was recovered in *fractions* 9–14 of the glycerol gradient with *fractions* 11–13 comprising the peak (Figure 4D). The analysis showed that the stoichiometry state of the Mcm10–Mcm2–7 complex is ∼ 5:1. This result indicates that five Mcm10 molecules would bind to each Mcm2–7 hexamer. This is consistent with our GST pulldown data in which Mcm10 may able to interact with every Mcm2–7 subunit with the exception of Mcm6. The stoichiometry of Mcm10 to Mcm2–7 *in vivo* was not determined in this study, and it may be different from our *in vitro* calculation.

### Mcm10 recruits Cdc45 to the Mcm2–7 complex

**Figure 5.**
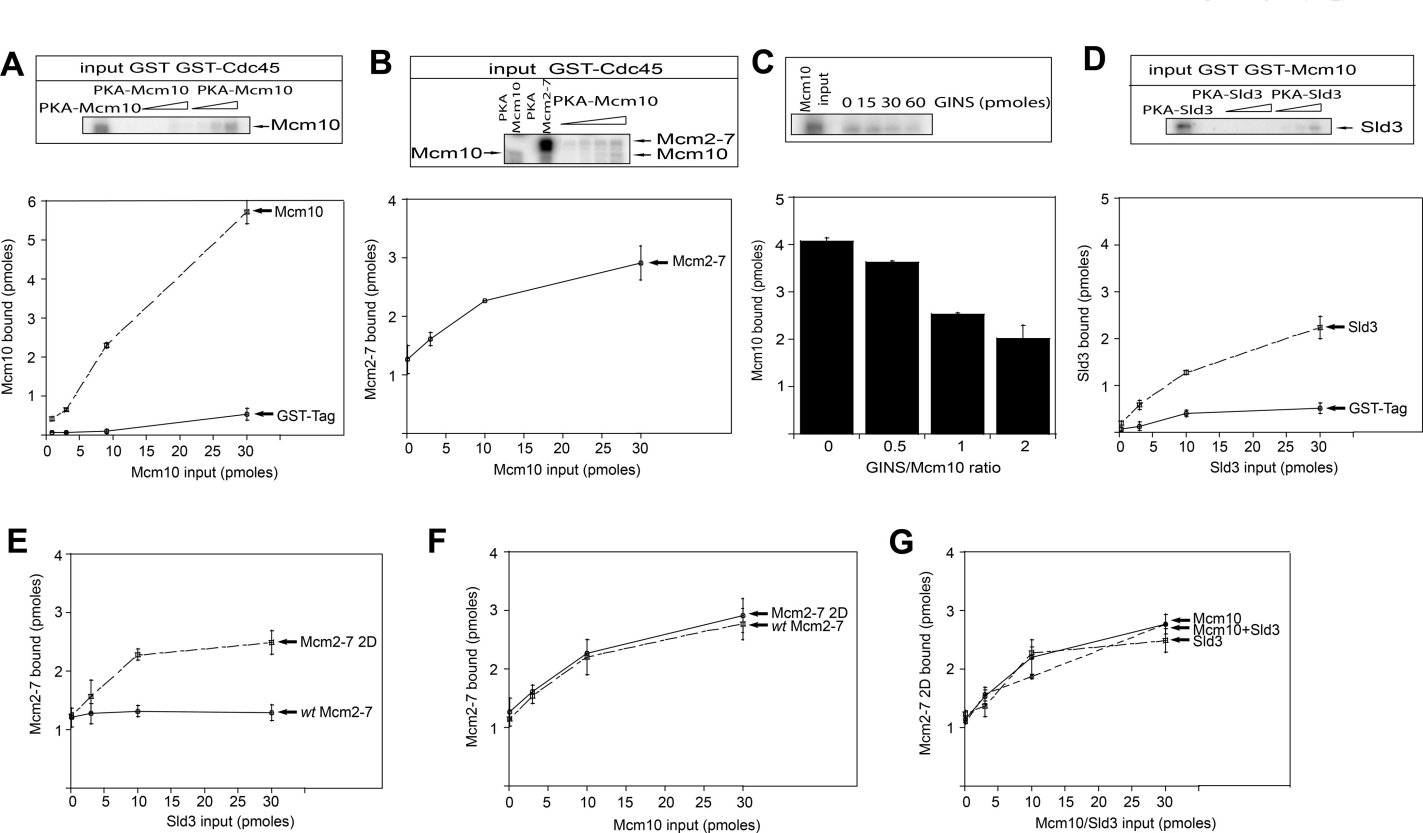
Mcm10 and Sld3 are two essential proteins involved in Cdc45 recruitment to the Mcm2–7 complex. (**A**) 30 pmol of GST tag or GST-Cdc45 was incubated with varying amounts of radiolabeled PKA-Mcm10 in a GST pulldown assay. Results from similar experiments were quantified, averaged and plotted. (**B**) 30 pmol of GST-Cdc45 was incubated with 10 pmol of radiolabeled PKA-Mcm2–7 and increasing amounts of radiolabeled PKA-Mcm10 in a GST pulldown assay. Results from similar experiments were quantified, averaged and plotted. (**C**) 30 pmol of GST-Cdc45 was incubated with 30 pmol of radiolabeled PKA-Mcm10 and varying amounts of GINS in a GST pulldown assay. Results from similar experiments were quantified, averaged and plotted. (**D**) 30 pmol of GST tag or GST-Mcm10 was incubated with varying amounts of radiolabeled PKA-Sld3 in a GST pulldown assay. Results from similar experiments were quantified, averaged and plotted. (**E**) 30 pmol of GST-Cdc45 was incubated with 10 pmol of either radiolabeled PKA-Mcm2–7 or PKA-Mcm2–7 2D and increasing amounts of radiolabeled PKA-Sld3 in a GST pulldown assay. Results from similar experiments were quantified, averaged and plotted. (**F**) 30 pmol of GST-Cdc45 was incubated with 10 pmol of either radiolabeled PKA-Mcm2–7 or PKA-Mcm2–7 2D and increasing amounts of radiolabeled PKA-Mcm10 in a GST pulldown assay. Results from similar experiments were quantified, averaged and plotted. (**G**) 30 pmol of GST-Cdc45 was incubated with 10 pmol of radiolabeled PKA-Mcm2–7 2D and increasing amounts of radiolabeled PKA-Mcm10, PKA-Sld3 or Mcm10-PKA and Sld3-PKA in a GST pulldown assay. Results from similar experiments were quantified, averaged and plotted. Graphs from (A), (B), (C), (D), (E), (F) and (G) represent mean values from two independent experiments and error bars indicate the standard deviation of the mean.

Mcm10 binds to Mcm2 with highest affinity compared to other subunits of the Mcm2–7 complex (Figure 4C). It is also known that Cdc45 interacts with the N-terminal domain of Mcm2 for CMG complex assembly (12). In order to investigate whether Mcm10 has a role in recruiting Cdc45 to the Mcm2–7 complex, we first determined if Mcm10 is able to bind directly to Cdc45. GST-Cdc45 was incubated with varying amounts of radiolabeled PKA-Mcm10 and a direct interaction was observed by GST pulldown assay (Figure 5A). To test if Mcm10 can recruit Cdc45 to the Mcm2–7 complex, we next incubated GST-Cdc45 with the radiolabeled PKA–Mcm2–7 and increasing amounts of radiolabeled PKA–Mcm10. We found a direct interaction between Cdc45 and Mcm2–7 (Figure 5B) as expected. As increasing concentrations of Mcm10 were added to the reaction, the amount of Cdc45 bound to Mcm2–7 substantially increased (Figure 5B). Since both the Mcm2–7 complex and Mcm10 are radiolabeled in this experiment, we observe a simultaneous increase in the binding of both Mcm10 and Mcm2–7 to Cdc45 (Figure 5B). These data suggest that Mcm10 can recruit Cdc45 to the Mcm2–7 complex *in vitro*. During S phase, GINS interacts with Cdc45 to form the active CMG complex (6,15,16,55). To determine if GINS is able to disrupt the interaction between Mcm10 and Cdc45, we incubated GST-Cdc45 with radiolabeled Mcm10 and increasing amounts of GINS. GST-Cdc45 pulls down a substantial fraction of Mcm10 as shown before (Figure 5A). As the concentration of GINS was increased, the level of Mcm10 bound to Cdc45 decreased (Figure 5C). These data indicate that GINS inhibits the interaction between Cdc45 and Mcm10 in a concentration-dependent manner, suggesting that Mcm10 and GINS may be competing with one another for the same binding site on Cdc45. The arrival of GINS at replication origins during S phase may help release Mcm10 from Cdc45.

### Different DDK requirement for Cdc45 recruitment by Mcm10 and Sld3

Previous studies showed that Sld3 helps recruiting Cdc45 to the Mcm2–7 complex (9,52,56). Our *in vivo* and *in vitro* data indicate that Mcm10 is also involved in the recruitment of Cdc45 to the Mcm2–7 complex. First, we wanted to study the interaction between Mcm10 and Sld3. We purified these proteins and studied their interaction *in vitro* using a GST pulldown assay. A weak interaction was detected between Mcm10 and Sld3 (Figure 5D).

Helicase activation is initiated by DDK which phosphorylates the Mcm2–7 double hexamer. DDK promotes the interaction between Cdc45 and Sld3 with the Mcm2–7 complex (39,57–60). Previous data showed that DDK phosphorylates two serine residues at the N terminus of Mcm2, Ser164 and Ser170, and that this phosphorylation is required for yeast growth (50). These residues were mutated to Asp (Mcm2 2D) in order to create a phosphomimetic mutant that resembles the DDK-phosphorylated version of Mcm2. We wanted to study if the phosphorylation of Mcm2 by DDK during S phase is sufficient to promote the binding of Sld3 and Cdc45 to the Mcm2–7 complex *in vitro*. The *wild-type* and mutant Mcm2–7 complexes were reconstituted from individually proteins using either Mcm2 or Mcm2 2D. We performed a recruitment assay in which we incubated GST-Cdc45 with either radiolabeled *wild-type* Mcm2–7 or Mcm2–7 2D and increasing amounts of radiolabeled Sld3. We found a direct interaction between Cdc45 and both *wild-type* Mcm2–7 and Mcm2–7 2D (Figure 5E). As increasing concentrations of Sld3 were added to the reaction, the amount of Cdc45 bound to Mcm2–7 2D substantially increased (Figure 5E). In contrast, when we performed the same experiment using *wild-type* Mcm2–7, the amount of Cdc45 bound to the Mcm2–7 complex remain the same even at the highest concentration of Sld3 (Figure 5E). These data indicate that the recruitment of Cdc45 to Mcm2–7 by Sld3 is DDK-dependent and that the phosphorylation at residues Ser164 and Ser170 of Mcm2 by DDK is sufficient to drive the interaction between Sld3 and Cdc45 with the Mcm2–7 complex *in vitro*. We next determined whether Mcm10 is able to recruit Cdc45 to Mcm2–7 2D. We incubated GST-Cdc45 with radiolabeled Mcm2–7 2D and increasing amounts of radiolabeled Mcm10. We found that Mcm10 can also recruit Cdc45 to Mcm2–7 2D *in vitro* (Figure 5F). The amount of Cdc45 recruited by Mcm10 was the same when we used either Mcm2–7 or Mcm2–7 2D (Figure 5F). Thus, Sld3 recruits Cdc45 in a DDK-dependent manner, whereas Mcm10 is able to recruit Cdc45 with no phosphorylation of Mcm2 by DDK. In order to investigate if Mcm10 and Sld3 cooperate during the recruitment of Cdc45 to DDK-phosphorylated Mcm2–7 complex, we incubated GST-Cdc45 with radiolabeled Mcm2–7 2D and increasing amounts of radiolabeled Mcm10, Sld3 or Mcm10 and Sld3 together. We found that the recruitment of Cdc45 to Mcm2–7 2D was similar in the presence of Sld3, Mcm10 or both together (Figure 5G).

### DDK phosphorylation of Mcm2 promotes Cdc45 recruitment to Mcm2–7 by Sld3

**Figure 6.**
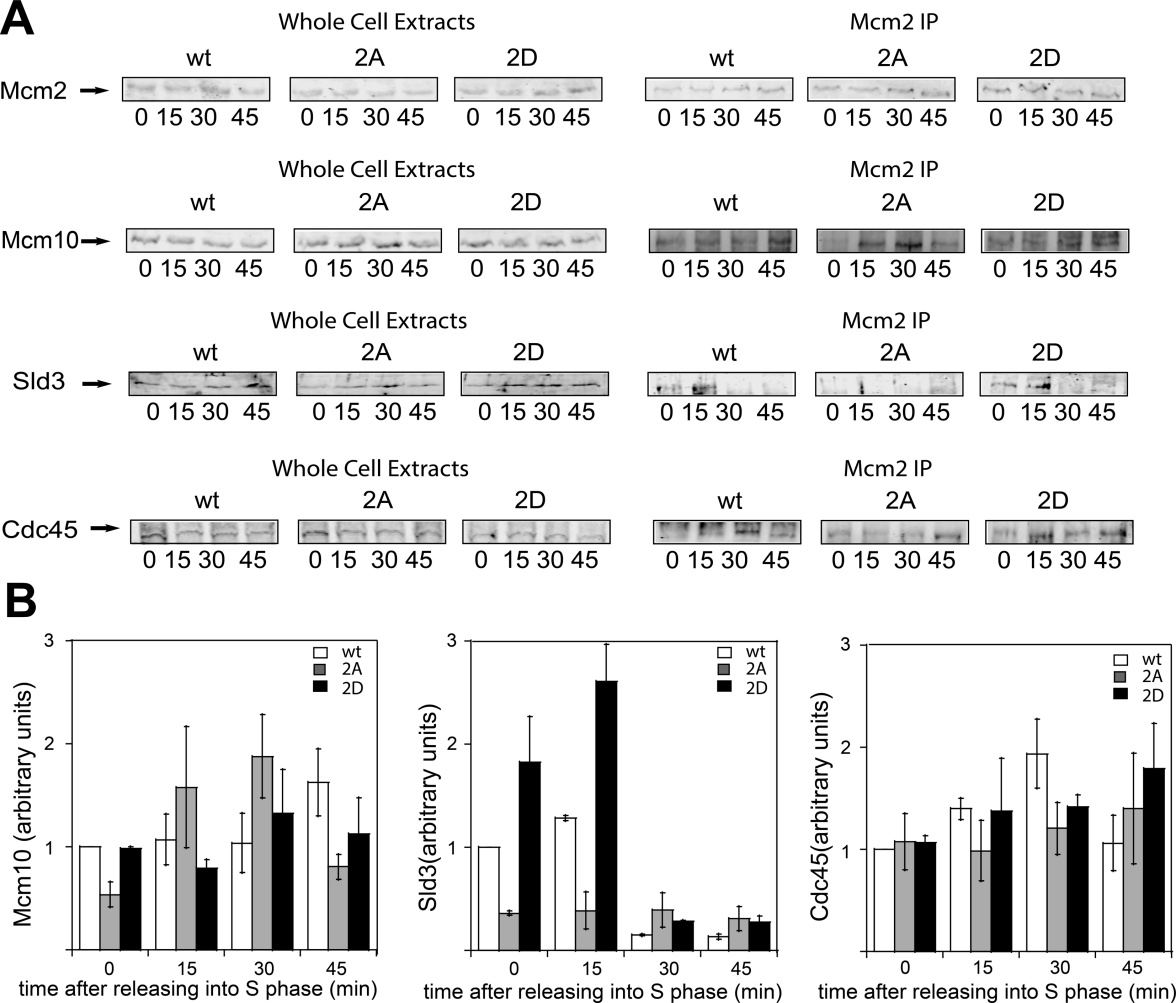
DDK phosphorylation of Mcm2 is required for Cdc45 recruitment to Mcm2–7 by Sld3. *mcm2-*td cells expressing *wild-type mcm2, mcm2–2A and mcm2–2D* were grown as described in Material and Methods. (**A**, **left panel**) Whole cell extracts were analyzed by western blot for the expression of the indicated proteins. (**A**, **right panel**) Cells were immunoprecipitated with antibodies directed against Mcm2, followed by western analysis with antibodies directed against Mcm2, Mcm10, Sld3 and Cdc45. (**B**) Results from experiments similar to those shown in (A) were quantified, averaged and plotted. Graphs represent mean values from two independent experiments and error bars indicate the standard deviation of the mean.

To study the effect of DDK phosphorylation of Mcm2 on Sld3- or Mcm10-mediated recruitment of Cdc45 to Mcm2–7 *in vivo*, we performed additional studies. For that purpose, we used a published strain for conditional temperature-induced degradation of endogenous *MCM2* gene (*mcm2-td* strain) (44). We transformed these cells with our galactose-inducible *wild-type mcm2, mcm2–2A (mcm2-S164A-S170A)* and *mcm2–2D (mcm2-S164D-S170D)* genes under control of the GALS promoter, a system with low expression as compared with other GAL promoters. At 25ºC in the absence of galactose, the endogenous *MCM2* gene is expressed. At 37°C in the presence of galactose, the *wild-type mcm2, mcm2–2A and mcm2–2D* genes are expressed, whereas the native *wild-type* Mcm2 protein is degraded. Cells were arrested in G_1_ with α-factor and then released into medium lacking α-factor for 0, 15, 30 and 45 min at the restrictive temperature (Figure 6).

Whole cell extracts exhibited similar levels of Mcm2, Mcm10, Cdc45 and Sld3 suggesting that equivalent levels of protein are expressed *in vivo* in cells expressing *wild-type mcm2*, *mcm2–2A* and *mcm2–2D* (Figure 6A, left panel). In co-IP analysis with antibodies directed against Mcm2, equivalent levels of Mm2 are observed. We probed Mcm2 immunoprecipitates with Mcm10 antibody and we detected Mcm10 bound at 15 and 30 min in cells expressing *mcm2–2A* or *mcm2–2D*, indicating that the binding of Mcm10 with Mcm2–7 is not affected by the phosphorylation of Mcm2 by DDK (Figure 6A, right panel). This result supports the idea that the interaction of Mcm10 with the Mcm2–7 complex during S phase is DDK-independent. When we probed immunoprecipitates with Sld3 antibody, we found that Sld3 is able to bind to Mcm2–7 in G_1_ and early S phase in cells expressing *wild-type mcm2* and *mcm2–2D*. In contrast, we are not able to detect Sld3 signal in the presence of *mcm2–2A* (Figure 6A, right panel), indicating that Sld3 binding to Mcm2–7 is DDK-dependent and that mutation at residues S164 and S170 of Mcm2 is sufficient to prevent this binding. We next probed the immunoprecipitates with Cdc45 antibody and found that the amount of Cdc45 bound to Mcm2–7 at 30 min after entry into S phase in cells expressing *mcm2–2A* is slightly reduced when compared with cells expressing *wild-type mcm2*. This indicates that, in the absence of Mcm2 phosphorylation by DDK, Cdc45 recruitment by Sld3 is inhibited. On the other hand, we can still detect some Cdc45 signal at 15 and 30 min in cells expressing *mcm2–2A* (Figure 6A, right panel). This weak interaction between Cdc45 and Mcm2–7 may be related to the observation that Mcm10 may recruit Cdc45 to Mcm2–7 in an Sld3 and DDK-independent manner.

## DISCUSSION

We found using purified proteins that Mcm10 binds directly to the Mcm2–7 complex and Cdc45. Furthermore, Mcm10 can recruit Cdc45 to the Mcm2–7 complex *in vitro* in a DDK-independent process. Sld3 also participates in a DDK-dependent mechanism in which the phosphorylation of the N-terminal region of Mcm2 by DDK is sufficient for Cdc45 recruitment to the Mcm2–7 complex. We also found severely defective DNA replication when Mcm10 is depleted *in vivo* using auxin-inducible protein degradation. The interaction of both helicase coactivators Cdc45 and GINS with the Mcm2–7 complex is delayed in the absence of Mcm10 *in vivo*. In addition, both Mcm2 phosphorylation by DDK and RPA interaction to origins of replication are reduced in the absence of Mcm10, indicating an essential role of Mcm10 in stimulating DDK phosphorylation of Mcm2 to promote origin melting.

### Mcm10 interacts with the loaded Mcm2–7 complex

Mcm2–7 complex is loaded in an inactive form at origins of replication during late M and G_1_ phase of the cell cycle (1,3). Loaded Mcm2–7 is then activated during S phase by recruiting Cdc45 and GINS to form the active CMG helicase (55). Mcm10 binds to origins of replication at the G_1_/S phase transition (61) and it is believed to have an essential role during helicase activation (19). We show here that Mcm10 interacts directly with the Mcm2–7 complex *in vitro* showing a ∼5:1 stoichiometry (Figure 4). However, we do not know the stoichiometry of Mcm10 for Mcm2–7 *in vivo*. Furthermore, we show an interaction with the Mcm2–7 complex during S phase *in vivo* (Figure 1C), indicating that Mcm10 binds to chromatin-loaded Mcm2–7 as it was described before (28,42).

### Mcm10 is required for timely assembly of the CMG complex

Activation of the replicative helicase is a key step for the initiation of DNA replication. This activation involves the assembly of the CMG complex during S phase (6,7). Cdc45 binds weakly to the Mcm2–7 complex *in vitro* in the absence of additional factors (52), indicating that a recruiting protein may be necessary to the association of Cdc45 with the Mcm2–7 complex. Previous *in vivo* data suggest that Sld3 is responsible for the recruitment of Cdc45 to Mcm2–7 complex (56). We show in this manuscript an additional mechanism in which Mcm10 can also participate in Cdc45 recruitment *in vivo*. First, we demonstrate that the binding of Cdc45 to the Mcm2–7 complex during S phase is delayed in the absence of Mcm10 *in vivo* (Figure 2 and Supplementary Figure S1). Then, we showed that Mcm10 interacts directly and recruits Cdc45 to the Mcm2–7 complex *in vitro* (Figure 5A and B). These results are consistent with previously published data, suggesting that the role of Mcm10 in Cdc45 recruitment may be conserved in multiple organisms like budding and fission yeast (36,37) and even in higher eukaryotes like *Xenopus* (28) and humans (38). This report disagrees with other recent reports suggesting that Mcm10 is not required for Cdc45 assembly with Mcm2–7 (40–42). Some of these other reports used ChIP, which involves fixing the cells. ChIP identifies Cdc45 that is in the vicinity of the origin, but does not determine whether Cdc45 is directly bound to Mcm2–7. Furthermore, these studies used 20 or 30 minutes as the S phase time point, which is later in S phase. The 15 min time point that we use is assessing CMG formation during early S phase. Finally, some of these studies used temperature-sensitive degrons instead of auxin-inducible degrons and the results may be temperature-sensitive. This may explain the discrepancy in our results.

When we analyzed CMG complex assembly *in vivo*, we also detected a delayed GINS binding to the Mcm2–7 complex in cells lacking Mcm10 (Figure 2 and Supplementary Figure S1). This result demonstrates that Mcm10 is also required for timely GINS association with Mcm2–7 as described before in human cells (38) and it contradicts other reports suggesting that Mcm10 is not required for GINS association with Mcm2–7 (40–42). We attribute our novelty to the fact that we are the first to use a 15 min time point for early S phase, no fixing of cells and 25°C, unlike previous reports.

At later time points during S phase, we detected that the binding of both Cdc45 and GINS with Mcm2–7 is unaffected in cells depleted for Mcm10. It is possible that residual Mcm10 molecules are executing this function at later time points. Also, Sld3 may be slowly stimulating DDK phosphorylation of Mcm2 in the absence of Mcm10 at later time points. Furthermore, Sld3 may be slowly recruiting Cdc45 to Mcm2–7 in the absence of Mcm10 at later time points (this will be discussed further below).

### Mcm10 and Sld3 are two essential recruitment proteins for Cdc45 attachment to the Mcm2–7 complex

**Figure 7.**
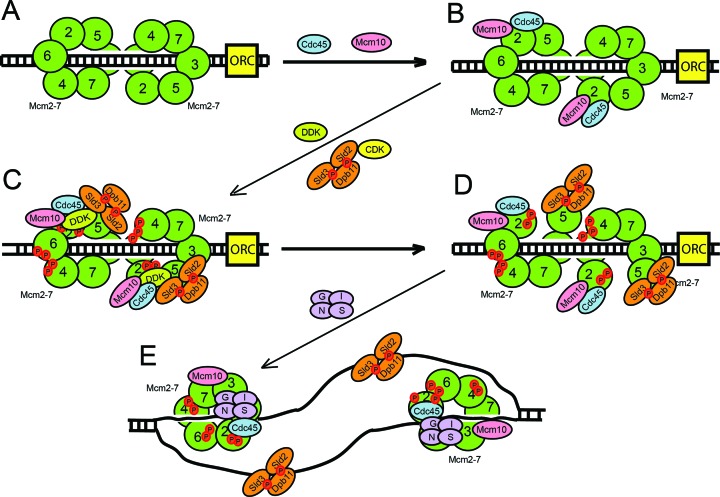
Mcm10 regulates the assembly of the CMG complex and DNA origin melting. (**A**) Mcm2–7 complex is loaded as a double hexamer to encircle dsDNA during late M and G_1_ phase. (**B**) In early S phase, Mcm10 interacts with the Mcm2–7 complex and recruits Cdc45 to Mcm2–7. Five molecules of Mcm10 may bind to each Mcm2–7, but only one Mcm10 is shown for clarity. (**C**) Mcm10 stimulates DDK phosphorylation of Mcm2. Sld3 also stimulates DDK phosphorylation of Mcm2, and Sld3 can also recruit Cdc45 in a manner that depends upon DDK phosphorylation of Mcm2. Sld3 has been recently shown to form a heterotetramer with Sld7 (68), but just one Sld3 molecule is shown for clarity. S-phase cyclin dependent kinase (CDK) phosphorylates Sld2 and Sld3 and these phosphorylated proteins bind to Dpb11 to form the ternary complex Sld3–Sld2–Dpb11 that binds to the Mcm2–7 complex. The Sld3–Sld2–Dpb11 ternary complex blocks the interaction between GINS and Mcm2–7. (**D**) Phosphorylation of Mcm2 by DDK opens the Mcm2–Mcm5 gate, allowing the extrusion of ssDNA from the central ring of Mcm2–7 complex. (**E**) Sld3-Sld2-Dpb11 binds to ssDNA and GINS binds to the Mcm2–7-Cdc45 complex, releasing Mcm10 from Cdc45 and forming the closed Cdc45–Mcm2–7–GINS active helicase complex that encircles ssDNA.

We show here that, in the absence of Mcm10, Cdc45 recruitment to the Mcm2–7 complex is delayed from 15 minutes to 30 minutes after release from α-factor arrest (Figure 2 and Supplementary Figure S1). This is a very substantial delay, considering DNA replication is half-complete by 30 minutes. These data suggest that Mcm10 is required for the timely association of Cdc45 with the Mcm2–7 complex *in vivo*. Furthermore, we show that Mcm10 recruits Cdc45 to Mcm2–7 *in vitro* and *in vivo* in the absence of DDK activity, suggesting that this mechanism is DDK-independent (Figures 5B and 6). Previous studies have shown that Sld3 can recruit Cdc45 to Mcm2–7 by a DDK-dependent mechanism (39,57–60). Our *in vitro* and *in vivo* data support these studies in which Sld3 recruitment of Cdc45 to Mcm2–7 is DDK-dependent (Figures 5E and 6). Furthermore, we may have elucidated the mechanism: DDK phosphorylation of Mcm2 stimulates Sld3 recruitment of Cdc45 to the Mcm2–7 complex. Taken together, these data suggest that there are two essential recruitment proteins for Cdc45 attachment to the Mcm2–7 complex: Mcm10, which acts early and Sld3, which depends upon DDK phosphorylation of Mcm2 (Figure 7). It may be critical to have both Sld3 and Mcm10 required for the timely Cdc45 recruitment to Mcm2–7 to ensure that both Sld3 and Mcm10 are present at Mcm2–7 for subsequent initiation steps. The requirement may be based upon a necessity for Mcm10 at Mcm2–7 for Pol α-primase stability (45,61), and the necessity for Sld3 at Mcm2–7 for the subsequent recruitment of GINS to Mcm2–7 (62).

### Mcm10 is required for origin DNA melting by stimulating DDK phosphorylation of Mcm2

Recent work demonstrated that Mcm2 phosphorylation by DDK during S phase may be responsible for opening the Mcm2–Mcm5 gate allowing the extrusion of the lagging strand from the Mcm2–7 ring (14). Under conditions where Mcm10 is depleted, the association of RPA to origins of replication is reduced (Figure 1D and E), suggesting that Mcm10 is involved in origin melting and ssDNA extrusion from the Mcm2–7 ring. This result has been obtained in three different *in vivo* studies in budding and fission yeast (40–42), suggesting that the critical role of Mcm10 for origin DNA melting is conserved in eukaryotes. We show here that the amount of phosphorylated-Mcm2 during S phase is reduced to nearly half in the absence of Mcm10 *in vivo* (Figure 3A). We also demonstrate that Mcm10 interacts with the Dbf4 subunit of DDK and it is able to stimulate Mcm2 phosphorylation by DDK *in vitro* (Figure 3B). This is consistent with *in vitro* studies in which RPA association to origins of replication require DDK and Mcm10 (59). Previous studies in fission yeast demonstrated that the homologue of Mcm10 (Cdc23p) also stimulated the phosphorylation of the Mcm2–7 complex by the homologue of DDK (Dfp1-Hsk1 kinase) *in vitro* (63). Cdc23p also interacts with Dfp1-Hsk1 kinase (specifically with the Dfp1 subunit, which is the homologue of Dbf4) and with the Mcm2–7 complex *in vitro*. This interaction may be responsible for the recruitment of Dfp1-Hsk1 kinase to the Mcm2–7 complex and for the stimulation of Mcm2–7 phosphorylation (63). Our results indicate that Mcm10 could have an essential role during origin melting by stimulating DDK phosphorylation of Mcm2 (Figure 7). We demonstrate *in vitro* a direct role for Mcm10 in recruiting DDK to Mcm2; however, we cannot rule out that *in vivo*, Mcm4 may play an indirect role in the Mcm10-dependent stimulation of DDK phosphorylation of Mcm2.

The lack of DNA melting at origins of replication in the absence of Mcm10 could explain why GINS interaction to the Mcm2–7 complex is delayed during S phase in cells in which Mcm10 has been depleted. According to our model, the generation of ssDNA at origins of replication occurs in S phase while Mcm10 is bound to the Mcm2–7 complex and after Cdc45 recruitment (Figure 7). Before DNA melting occurs, Sld3–Sld2–Dpb11 ternary complex is bound to Mcm2–7–Cdc45, blocking the binding of GINS to Mcm2–7–Cdc45 because Sld3–Sld2–Dpb11 and GINS compete with one another for Mcm2–7–Cdc45 binding (46,47,64,65). Once DDK phosphorylates Mcm2, stimulated by Mcm10, ssDNA is generated after the opening of the Mcm2–Mcm5 gate and Sld3–Sld2–Dpb11 switches from Mcm2–7 to ssDNA binding, leaving the Mcm3–Mcm5 binding site for GINS (46,47,52,65) (Figure 7). In cells lacking Mcm10, origin melting is defective and Sld3–Sld2–Dpb11 stays bound to Mcm2–7–Cdc45, blocking the binding of GINS to the Mcm2–7 complex.

### Role of Mcm10 and Sld3 in replication initiation

There is an excess of Mcm2–7 double hexamer complexes loaded onto the DNA during late M and G1 phase, relative to the number of Mcm2–7 complexes that are activated during S phase (43). Key to the activation of the limited number of Mcm2–7 complexes during S phase is the phosphorylation of Mcm2–7 by DDK and the association of Mcm10 and other initiation factors (Sld2, Sld3, Dpb11 and Sld7) with Mcm2–7. In this manuscript, we explain the mechanism that ensures that kinase phosphorylation is coincident with initiation factor binding to the Mcm2–7 complex. We show that Mcm10 is loaded onto Mcm2–7 independently of DDK, consistent with previous reports suggesting that Mcm10 is recruited to Mcm2–7 before DDK (61,66). Interaction of Mcm10 with the Mcm2–7 complex stimulates the DDK phosphorylation of Mcm2. Simultaneously, Mcm10–Mcm2–7 interaction stimulates Cdc45 binding to Mcm2–7. Since Mcm10 directly recruits Cdc45 to Mcm2–7 and Mcm10 also directly stimulates DDK phosphorylation of Mcm2, Mcm10 couples Cdc45 recruitment to Mcm2–7 with DDK phosphorylation of Mcm2. Sld3 also stimulates DDK phosphorylation of Mcm2 *in vitro* and *in vivo* (67), and Sld3 can recruit Cdc45 to Mcm2–7 *in vitro* (this manuscript) and *in vivo* (56). These data suggest that Mcm10 and Sld3 couple Cd45 recruitment to Mcm2–7 with DDK phosphorylation of Mcm2. DDK phosphorylation of Mcm2 may subsequently lead to Mcm2–7 ring opening, allowing for the extrusion of ssDNA from the central channel of Mcm2–7 and the subsequent association of GINS with Cdc45-Mcm2–7. Thus, the role of Mcm10 and Sld3 is to couple the association of Cdc45 with a given Mcm2–7 double hexamer with the DDK phosphorylation of the same Mcm2–7 double hexamer. This leads to origin melting and the association of GINS with Cdc45-Mcm2–7. Therefore, the binding of Mcm10 and Sld3 to Mcm2–7 may be part of a selection mechanism to activate a particular Mcm2–7 double hexamer complex for subsequent helicase activation.

## Supplementary Material

SUPPLEMENTARY DATA
