# Supplementary material for: Mcm10 coordinates the timely assembly and activation of the replication fork helicase
Source: Nucleic Acids Res. 2015 Nov 17;44(1):315–29. doi: 10.1093/nar/gkv1260 (PMC4705653; doi:10.1093/nar/gkv1260)
Supplement: SUPPLEMENTARY DATA [file supp_44_1_315__index.html]

Mcm10 coordinates the timely assembly and activation of the replication fork helicase — Mcm10 coordinates the timely assembly and activation of the replication fork helicase — SUPPLEMENTARY DATA 

# Mcm10 coordinates the timely assembly and activation of the replication fork helicase

## SUPPLEMENTARY DATA

- SUPPLEMENTARY DATA
