# Supplementary material for: Mcm10 coordinates the timely assembly and activation of the replication fork helicase
Source: Nucleic Acids Res. 2015 Nov 17;44(1):315–29. doi: 10.1093/nar/gkv1260 (PMC4705653; doi:10.1093/nar/gkv1260)
Supplement: SUPPLEMENTARY DATA [file supp_gkv1260_nar-02061-d-2015-File002.pdf]

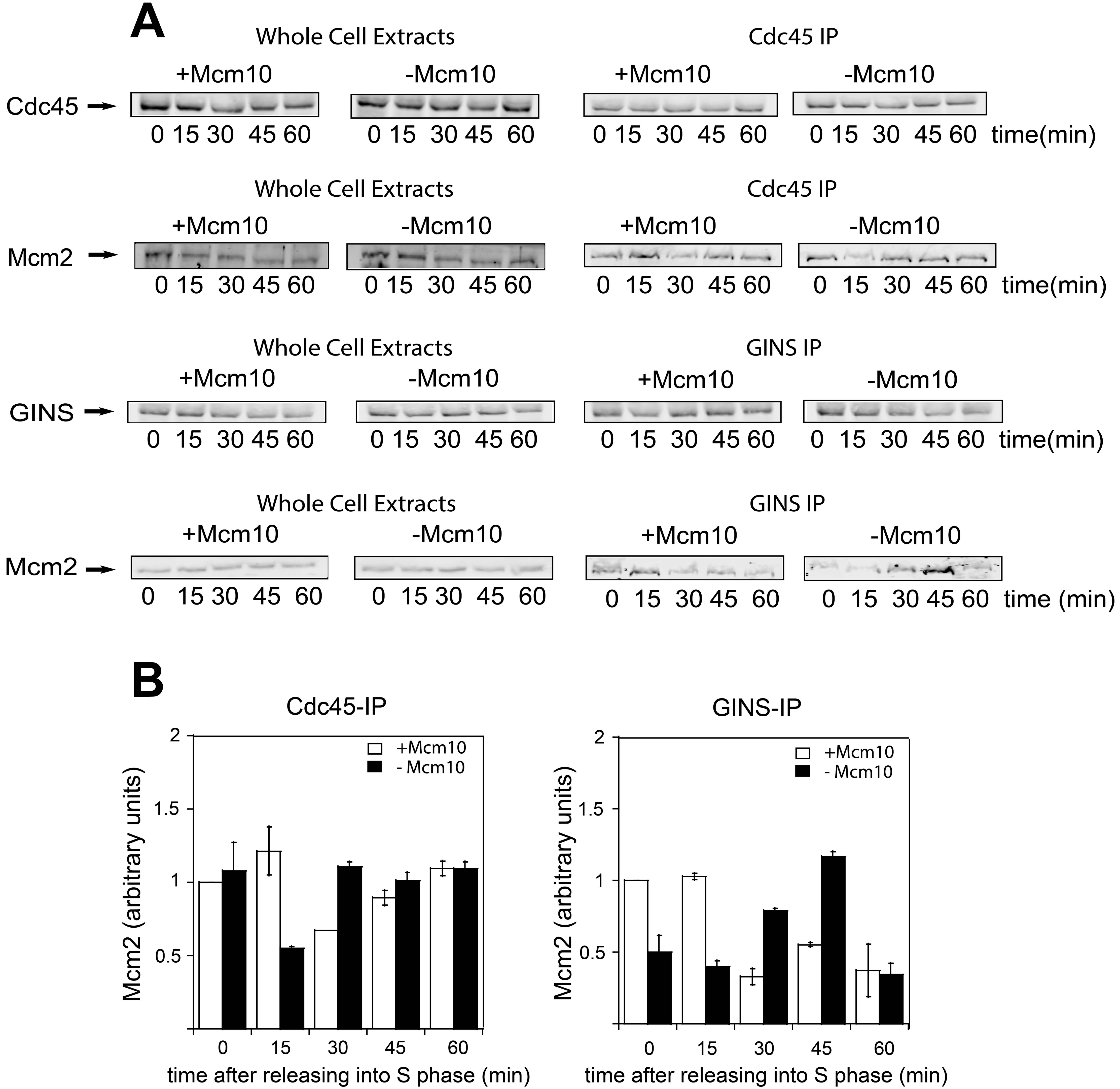
A
Whole Cell Extracts
Cdc45 IP
+Mcm10
-Mcm10
Cdc45
0 15 30 45 60
time(min)
Mcm2
GINS IP
GINS
time (min)
B
Cdc45-IP
GINS-IP
+Mcm10
- Mcm10
Mcm2 (arbitrary units)
2
1.5
1
0.5
0
15
30
45
60
time after releasing into S phase (min)

Supporting Material

Table S1, related to Material and Methods

Yeast strains used in this study

Strain	Genotipe	Source
BY25924	*MAT***a** *ura 3-1::GAL-OsTIR1-9MYC(URA3) mcm10::mcm10-1-aid (KanMX) cdc45::CDC45-5FLAG (hphNT) ade2-1 his3-11,15 leu2-3,112 trp1-1 ura3-1 can1-100*	Yeast Genetic Resource Center
BY25925	*MAT***a** *ura 3-1::GAL-OsTIR1-9MYC(URA3) mcm10::mcm10-1-aid (KanMX) psf2::PSF2-5FLAG (hphNT) MCM4::mcm4-6ha (k.l. TRP1) ade2-1 his3-11,15 leu2-3,112 trp1-1 ura3-1 can1-100*	Yeast Genetic Resource Center
BY25926	*MAT***a** *ura 3-1::GAL-OsTIR1-9MYC(URA3) mcm10::mcm10-1-aid (KanMX) rfa1::RFA1-5FLAG (HIS3) ade2-1 his3-11,15 leu2-3,112 trp1-1 ura3-1 can1-100*	Yeast Genetic Resource Center
YKL69	*MAT***a** *ade2-1 ura3-1 his3-11,15 trp1-1 leu2-3,112 can1-100 MCM2::mcm2-td (URA3) UBR1::GAL-ubiquitin-M-lacI fragment-Myc-UBR1(HIS3)*	Labib *et al*. (2000) Science

Figure S1. Delayed binding of Cdc45 and GINS to Mcm2-7 in the absence of Mcm10. A. *mcm10-1*-aid cells were grown as described in Material and Methods. **A, left panel.** Whole cell extracts were analyzed by Western blot for the expression of the indicated proteins. **A, right panel.** Same protocol was performed as the one used in Figure 2. We immunoprecipitated cells with antibodies directed against Cdc45 and GINS, followed by Western analysis with Mcm2 antibody. **B.** Results from experiments similar to those shown in (A) were quantified, averaged and plotted. Graphs represent mean values from two independent experiments and error bars indicate the standard deviation of the mean.
